# Energy Harvesting for TDS-OFDM in NOMA-Based Underwater Communication Systems

**DOI:** 10.3390/s22155751

**Published:** 2022-08-01

**Authors:** Hamada Esmaiel, Haixin Sun

**Affiliations:** 1 Department of Information and Communication, School of Informatics, Xiamen University, Xiamen 361005, China; h.esmaiel@aswu.edu.eg; 2 Electrical Engineering Department, Faculty of Engineering, Aswan University, Aswan 81542, Egypt

**Keywords:** underwater acoustic communication, NOMA, TDS-OFDM, energy harvesting, energy efficiency

## Abstract

Non-orthogonal multiple access (NOMA) is considered a promising multiple access technique for fifth generation (5G) mobile networks and tactical internet due to its high spectral efficiency. Thanks to the high spectral efficiency of NOMA, it can be a strong candidate suitable for the limited channel bandwidth of underwater acoustic communication. The NOMA transmitter is employing superposition coding (SC). The NOMA receiver is based on the successive interference cancellation (SIC) technique. The multicarrier NOMA adopts orthogonal frequency division multiplexing (OFDM) as a multicarrier modulation (MCM) technique; however, conventional cyclic prefix OFDM (CP-OFDM) and zero padding (ZP-OFDM) have inefficient spectral efficiency. Thanks to efficient synchronization and high energy-spectral efficiency of the time-division synchronization OFDM (TDS-OFDM), it is a significant attractive candidate for underwater multicarrier communication. However, wasting the power transmission of long guard intervals in the battery-based underwater communication is represented as one of the TDS-OFDM main drawbacks. Harvesting energy and improving the energy efficiency of acoustic-based TDS-OFDM-NOMA represent high achievement goal battery recharging challenges due to the ocean environment. This paper proposes time switching simultaneous wireless information and power transfer (TS-SWIPT) to harvest the energy of transmitted power over the guard interval in the TDS-OFDM-NOMA scheme. The proposed energy harvested scheme harvests the energy from the wasted power in the long guard interval and improves the energy efficiency of the TDS-OFDM multicarrier scheme. This study demonstrates the superiority of the proposed TDS-OFDM-NOMA over the underwater acoustic channel by revealing high energy efficiency, high spectral efficiency, better bit error rate performance, and high system data throughput.

## 1. Introduction

The underwater acoustic (UWA) channel is considered one of the most challenging channels due to its significant multipath spread, limited available bandwidth, poor physical link quality, and time-varied channels caused by Doppler effects. Therefore, multicarrier modulation is presented as an attractive solution to improve the utilization of the limited channel bandwidth and mitigate the wide delay spread of the underwater acoustic (UWA) channel. However, a significant Doppler’s effect presented in UWA communication leads to high inter-carrier interference (ICI) [1,2,3] in underwater multicarrier modulation. The most attractive multicarrier modulation technique used in UWA communication is orthogonal frequency division multiplexing (OFDM) due to its high capability of dealing with the UWA long multipath spread channel using low complex time-domain equalization technique used in single carrier communication systems [1,4,5]. Four schemes of OFDM are introduced for the UWA channels based on the guard interval insertion way: (1) cyclic prefix OFDM (CP-OFDM) [4], where the last part of the symbols in the transmitted signal is added on ahead of the transmitted frame; (2) zero padding OFDM (ZP-OFDM) [6] in this scheme, where the CP part is replaced by zeros; (3) time-domain synchronous OFDM (TDS-OFDM) [7], where the guard interval is used to transmit a pseudorandom noise (PN) training sequence known to both the transmitter and receiver; (4) the OFDM multicarrier technique without any guard intervals [1]. Each one of these multicarriers has its pros and cons. The CP-OFDM uses long pilots and guard intervals, and both are wasted on the spectral and energy of the UWA communication system. On the other side, CP insertion leads to a circular convolution instead of a linear convolution between the transmitted signals and the communication channel. Hence, such circular convolution relaxes the equalization. The ZP-OFDM was the preferred multicarrier communication technique in the UWA systems as it saves the power used in the guard interval transmission spatially, as the UWA communication systems are battery-based. However, ZP-OFDM is still suffering from the wasted spectral efficiency in the guard interval and pilots’ signals; plus, it suffers from the on-off hardware problem. Thanks to efficient synchronization and high energy/spectral efficiency, the TDS-OFDM system has been proposed for underwater communication, but it is suffering from the inter-block interference (IBI) problem due to the mutual interference between the training sequence used as a guard interval and OFDM data blocks. The no guard interval OFDM (NGI-OFDM) is an OFDM scheme without any guard intervals proposed for UWA communication to improve the channel utilization [1], but the NGI-OFDM scheme wastes high energy in the pilot’s needs for equalization, as it wastes half of the signal energy used in pilots. Moreover, it cannot provide a reliable communication system for the underwater channels due to the Doppler estimation task without any guard interval becoming a complicated task dominating the complexity of the receiver [8].

Recently, the non-orthogonal multiple access (NOMA) was proposed for the fifth-generation (5G) mobile network to increase spectral efficiency [9]. The NOMA spectral efficiency is compatible with the existing and future wireless communication networks. The conventional NOMA is a power domain system under the principles of superposition coding (SC) and successive interference cancellation (SIC) at the transmitter and receiver sides, respectively [10]. Compared to the orthogonal multiple access (OMA), where the number of users is limited due to the exclusivity of orthogonal resources, NOMA allows the allocation of the same orthogonal resources to more than one user simultaneously. The NOMA scheme was proposed for underwater communication to improve the utilization of the limited UWA channel [11]. In this paper, NOMA is adopted with OFDM and multiple users to allocate on a subcarrier at the same time to maximize the channel utilization. In this paper, the adoption of the NOMA-TDS-OFDM will be addressed and presented for underwater communication to obtain a reliable underwater communication system.

Thanks to the simultaneous wireless information and power transfer (SWIPT), the RF signal was utilized in power transfer as well as decoding information [12] in 5G mobile networks based on the NOMA scheme. Varshney proposed the main idea of transmitting energy and information at the same time [12]. Based on the capacity-energy function, he provides a fundamental tradeoff performance for power transfer and simultaneous information. SWIPT behaviors in multi-input multiple-output (MIMO) were studied in [13,14,15]. In [13], the receivers are divided to be a group for decoding information, and the others are used for harvesting the energy from signals sent via a common transmitter. This idea was extended by considering the imperfect channel state information (CSI) at the transmitter side in [14]. Energy harvesting in MIMO relay systems was studied in [15] where different tradeoffs between the energy transfer and information rates were investigated. SWIPT for multiuser systems was studied in [16] with a receiver constraint for multiple access channels to achieve a maximum sum rate for harvesting relay in the multi-hop channel. SWIPT performance over a wireless communication network was studied in [16,17]. The hybrid network which overlaps with an uplink cellular network deployed randomly and uses the beacon’s power for mobile wirelessly recharge was addressed in [16]. Moreover, ref.[16] derived a tradeoff in network parameters under outage constraint. SWIPT in the cognitive radio networks was addressed in [17]. The nearby transmission of primary transmission is used for harvesting energy of the secondary user under outage constraints to maximize the secondary user system throughput.

Unlike terrestrial communications, acoustic signals are used as wireless information transmission (WIT) instead of RF signals. The acoustic-based OFDM needs a long guard interval to avoid intercarrier interference (ICI) in addition to inserting a long pilot’s signal to estimate the long-tap delay of the UWA channel. To save the battery power of the underwater nodes, recently researchers preferred the TDS-OFDM as a fit candidate for the underwater multicarrier modulation [7], especially after applying the compressive sensing for estimating the channel impulse response using a small free region of the guard interval unaffected by the inter-block interferences (IBIs) among the OFDM data blocks and guard interval training sequence [7]. In this paper, the corrupted part of the received PN sequence in the received NOMA-TDS-OFDM frame is used for energy harvesting. The long guard interval of the underwater TDS-OFDM helps in increasing the energy harvested using the proposed scheme. The time switching (TS) SWIPT (TS-SWIPT) [18] energy harvesting technique is applied for the proposed acoustic-based NOMA-TDS-OFDM underwater communication system. This paper can be summarized as follows:

1. This paper presents the TDS-OFDM-NOMA over the UWA channel for the first time according to the authors’ knowledge. TDS-OFDM-NOMA is proposed in this paper to improve energy and spectral efficiency.
2. A complete receiver architecture is proposed in this paper for the TDS-OFDM-NOMA. The proposed receiver architecture addresses the Doppler shift estimation and compensation problems plus the UWA channel estimation problem.
3. This paper proposes the time switching simultaneous wireless information and power transfer to harvest the energy of transmitted power over the guard interval in the TDS-OFDM-NOMA scheme.
4. We provide a detailed energy harvesting receiver architecture for the SWIPT in the proposed TDS-OFDM-NOMA.
5. The performance of the proposed TDS-OFDM-NOMA scheme is evaluated in terms of average system throughput, bit error rate analysis, and energy harvesting improvement based on the SWIPT. The system performance is conducted over a statistical UWA channel.

The rest of this paper is organized as follows. In Section 2, we overview the basics of the TDS-OFDM system model. The proposed TDS-OFDM-NOMA scheme is modeled in Section 3. The proposed energy harvesting scheme of the proposed TDS-OFDM-NOMA is addressed in Section 4. Section 5 discusses the detailed TDS-OFDM-NOMA receiver proposed for decoding information. Experimental results are presented in Section 6, and the paper’s conclusions are summarized in Section 7.

## 2. TDS-OFDM System Model

In this section, the basic properties of TDS-OFDM are reviewed for our discussion. TDS-OFDM is the key technology in the Chinese standard of digital terrestrial multimedia/broadcasting (DTMB) and digital television terrestrial broadcasting (DTTB) [19]. The TDS-OFDM was proposed to avoid sending the pilot signal used in both CP-OFDM, ZP-OFDM, and NGI-OFDM for energy and bandwidth efficiency. However, TDS-OFDM energy and spectrum efficiency can cause significant bit error rate (BER) performance deterioration due to IBIs between the training sequence and OFDM data blocks [20]. The dual pseudorandom noise OFDM (DPN-OFDM) [21] and zero-pseudorandom noise OFDM (ZPN-OFDM) [22] were proposed to simplify the equalization and avoid the IBIs between OFDM data blocks and the training sequence. Although high BER improvement is achieved using DPN-OFDM and ZPN-OFDM, they waste channel bandwidth by using guard intervals twice in DPN-OFDM and ZPN-OFDM, making these schemes unfit for the limited UWA channel bandwidth [7]. In [7], the authors discussed how to avoid the IBI by estimating a semi-perfect UWA CSI using the IBI-free region and mitigating UWA channel interference by using compressive sensing techniques.

**Figure 1:**
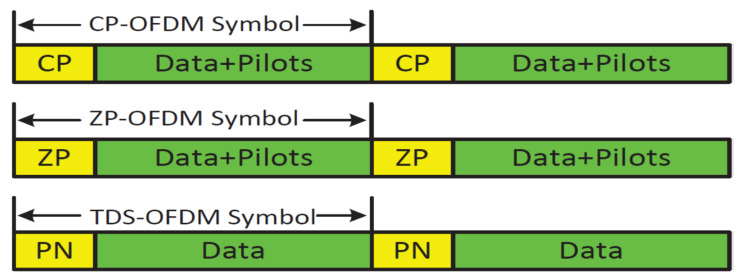
TDS-OFDM, ZP-OFDM and CP-OFDM signal structure comparison in the time domain.

The detailed comparison of frame structures among CP-OFDM, ZP-OFDM, and TDS-OFDM signals in time domains is shown in Figure 1, where the CP used by CP-OFDM is replaced by zeros in ZP-OFDM and by a known PN training sequence in TDS-OFDM. The frequency-domain symbol vector $s_{N}={\left[s_{0},s_{1},\cdots ,s_{N-1}\right]}^{T}$ is transmitted in the TDS-OFDM multicarrier system over the N subcarrier. The i-th time-domain transmission vector of the TDS-OFDM is:

(1) $$x_{i}={\left\{x_{i,k}\right\}}_{k=0}^{N-1}=F_{N_{i}}^{H}s_{N_{i}}$$

where $F_{N}$ is the N point fast Fourier transform (FFT) matrix with the $\left(m,n\right)$-th element $F_{N}\left(m,n\right)=\frac{1}{\sqrt{N}}e^{-j2\pi mn/N}$. k is the k-th element of the data block. The guard interval with length M is represented by $c_{M}={\left[c_{0},c_{1},\cdots ,c_{M-1}\right]}^{T}$ in the conventional TDS-OFDM scheme. The total transmitted TDS-OFDM frame is defined as:

(2) $${𝓼}_{i}={\left[{𝓼}_{i,0},{𝓼}_{i,1},\cdots \cdots ,{𝓼}_{i,M+N-1}\right]}^{T}={\left[x_{i},c_{i}\right]}_{M+N-1}^{T}$$

Let the transmitted PN sequence of the TDS-OFDM be ${\left\{c_{i,q}\right\}}_{q=0}^{M-1}$. The TDS-OFDM transmitted symbol is denoted as $a\left(n\right)$ and can be expressed as:

(3) $$a\left(n\right)=\left\{\begin{array}{c} \frac{1}{N}\sum_{k=0}^{N-1}S\left(k\right)e^{j2\pi kn/N}\;\;n\in \left[0,N-1\right]\\ {\left\{c_{i,k}\right\}}_{k=0}^{M-1}\;n\in \left[N,N+M-1\right] \end{array}\right.$$

**Figure 2:**
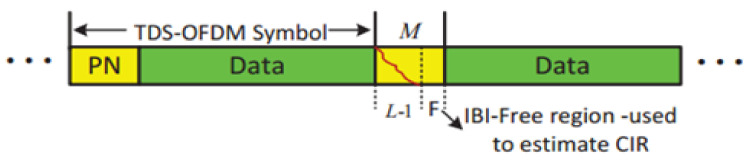
TDS-OFDM, the received TDS-OFDM frame.

This TDS-OFDM frame is transmitted over the UWA multi-path channel. The channel can be modeled by a quasi-static $L_{ch}$-th order finite impulse response (FIR) filter with channel impulse response (CIR) ${\left\{h_{i,k}\right\}}_{k=0}^{L_{ch}-1}.$ Assume the length of M is larger than the maximum channel tap delay, i.e., $M\geq L_{ch}.$ The *i*-th received OFDM frame ${\left\{r_{i,k}\right\}}_{k=0}^{N+M-1}$ consists of two parts; (1) $\;{\left\{x_{i,k}\right\}}_{k=0}^{N+L_{ch}-1},\;i\geq 0$, indicating the convolution between the information sequence ${\left\{s_{i,k}\right\}}_{k=0}^{N-1}$ and the CIR; (2) ${\left\{d_{i,k}\right\}}_{k=0}^{M+L_{ch}-1}=0,\;i\geq 0$ indicating the linear convolution between the PN sequence ${\left\{c_{i,k}\right\}}_{k=0}^{M-1}=0,\;i\geq 0,$ and the CIR. In this regard, the expressions of these parts are given by:

(4) $$x_{i,k}=s_{i,k}⊕h_{i,k}=\sum_{l=0}^{L_{ch}-1}s_{i,k-1}\cdot h_{i,l},\;0\leq k<N+L_{ch}-1$$

(5) $$d_{i,k}=c_{i,k}⊕h_{i,k}=\sum_{l=0}^{L_{ch}-1}c_{i,k-1}\cdot h_{i,l},\;0\leq k<M+L_{ch}-1$$

where ⊕ is the linear convolution operation. The received signal frame ${\left\{r_{i,k}\right\}}_{k=0}^{N+M-1}$ can be expressed as:

(6) $$r_{i,k}=u_{i,k}+n_{i,k}$$

where,

(7) $$u_{i,k}=\left\{\begin{array}{c} d_{i-1,k+N}+x_{i,k}\;0\leq k<L_{ch}-1\\ x_{i,k}\;L_{ch}-1\leq k<N+L_{ch}-1\\ d_{i,k}\;N\leq k<N+M-1 \end{array}\right.$$

and $n_{i,k}$ is the channel additive white Gaussian noise (AWGN). Figure 2 shows the corrupted received sequence; in the received TDS-OFDM frame, the guard interval would affect the OFDM data part, and the tail of OFDM data spreads into the guard interval of the next OFDM data block. The IBI between the OFDM data blocks and its PN sequence contains different features, and, as the OFDM data blocks are unknown, it is hard to obtain perfect estimation over the UWA channel. Completely removing the occurring IBI is a hard job, where OFDM data blocks with large frame sizes are used in the UWA channel to improve spectral efficiency, but it increases the computation complexity of removing the IBIs that occurred in the PN training sequence. Thanks to compressive sensing theory and the sparsity nature of the UWA channel, the authors in [7] used a small IBI-free region for semi-perfect compressive sensing-based channel estimation. With semi-perfect channel estimation and known PN training sequence at the receiver side, the mutual interference of the OFDM data block can be removed, and the BER of the acoustic-based TDS-OFDM can be significantly improved.

## 3. TDS-OFDM-NOMA Scheme

**Figure 3:**
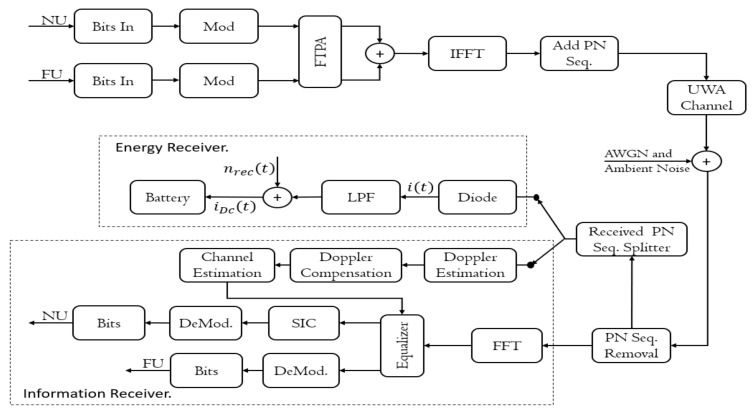
The TDS-OFDM-NOMA communication system.

In this section, the TDS-OFDM-based multi-carrier NOMA system in an underwater downlink scenario is presented. The functional block diagram of the multicarrier TDS-OFDM-NOMA is shown in Figure 3. For simplicity and without loss of generality, a single-input and single-output (SISO) system with one base station (BS) and two users as a minimum are used. In this paper, we assume the transmitter and receiver are operating with a single antenna. Near user (NU) is the unmanned underwater vehicle (UUV) close to the sink node (SN). Another UUV working at a far communication distance from the SN is recognized as a far user (FU). The near and far users are multiplexing in the power domain NOMA based on the user’s channel conditions. By pairing the near and far users, the NOMA scheme improves the total capacity of the system. NU has the better channel condition with low power $\left(P_{1}\right)$. The high power $\left(P_{2}\right)$ is assigned to the FU and with a bad channel condition. There are different power allocation schemes of NOMA that have been proposed in the literature [23]. In this paper, the fractional transmit power allocation (FTPA) with a condition, i.e., $P_{1}+P_{2}=1,$ is used for simplicity. On the transmitter side, bits streams from each user are converted to symbols by using the conventional mapper. The transmitted symbols are then followed by the FTPA for power allocation. $s_{1}$ and $s_{2}$ are the transmitted data from the NU and FU, respectively. In detail, as shown in Figure 3, the near and far user modulated bits are powered with different levels of power by using the FTPA scheme. After that, the NU signal and FU signal are combined. The combined signal is modulated over the OFDM conventional modulation using an inverse Fourier transform. The TDS-OFDM PN sequence was added for the modulated OFDM signal in the time domain. The TDS-OFDM frame is transmitted over the UWA channel in addition to adding the additive white Gaussian noise plus the ambient noise effect. On the receiver side, the first step is removing the PN sequence, and this removed sequence is divided into two parts; (1) uncorrupted part; (2) corrupted part. The uncorrupted part is used for Doppler estimation, channel estimation, and Doppler compensation. The corrupted part is used for energy harvesting using a rectifier diode and low pass filter to feed the harvested energy to the internet of the underwater node. The OFDM received frame will be demodulated using the OFDM demodulation system. In the OFDM demodulation system, the Doppler estimation, channel estimation, and Doppler compensation estimated using the uncorrupted part of the received PN sequence are used for equalization. After equalization, the FU demodulated its signal considering the NU signal as noise. For the NU, the SIC technique is used to estimate the information. In our underwater acoustic communication system, the transmitted symbols are multiplexed into a superimposed signal $s\left(t\right)$ through SC as follows:

(8) $$s\left(t\right)=\sqrt{P_{1}}s_{1}\left(t\right)+\sqrt{P_{2}}s_{2}\left(t\right)$$

The superimposed signal is then directed to the inverse IFFT. Let T denote the OFDM symbol duration, and $T_{g}$ is the duration of the guard interval. The total duration of the TDS-OFDM frame is $T=\overset{´}{T}+T_{g}$. The subcarrier frequency spacing is $\Delta f=\frac{1}{T}$. The *k*-th subcarrier will be at the frequency of $f_{k}=f_{c}+N\Delta f$; k=0,…, N. The multiplexing output signals of the IFFT are given as:

(9) $$x_{k}=\frac{1}{\sqrt{N}}\sum_{k=0}^{N-1}s\left(k\right)e^{j2\pi k\Delta ft}$$

where N is the IFFT size, and it represents the total number of orthogonal sub-carriers in the proposed UWA communication system. $f_{k}=f_{c}-\frac{B}{2}+\frac{k}{T_{s}}$, $f_{c}$ is the center frequency; $T_{s}$ is the symbol duration; and B=NTs represents the frequency bandwidth for the transmitted signal. In this paper, we use $f_{c}=3072\;\mathrm{Hz},$ N=1024 subcarrier, $T_{s}$ = 1 s, B=1024 Hz; hence, the subcarrier spacing is 1 Hz.

Unlike the conventional CP-OFDM and ZP-OFDM, where the data stream from the IFFT block is converted into the serial stream to append the cyclic prefix and prevent inter-symbol interference, the TDS-OFDM inserts a PN sequence as a guard interval. As in [24], in this paper, the PN training sequence is used for the frequency domain to relax the underwater Doppler estimation and multiplexing. The TDS-OFDM signals’ blocks can be written as:

(10) $$s\left(t\right)=\frac{1}{\sqrt{N}}\sum_{k=0}^{N-1}s\left(k\right)e^{j2\pi k\Delta ft}+\frac{1}{\sqrt{M}}\sum_{k=N}^{N+M}c\left(k\right)e^{j2\pi k\Delta ft}$$

The transmitted signal is then passed over the UWA channel in the passband as:

(11) $$s\left(t\right)=Re\left\{\left[\frac{1}{\sqrt{N}}\sum_{k=0}^{N-1}s\left(k\right)e^{j2\pi k\Delta ft}+\frac{1}{\sqrt{M}}\sum_{k=N}^{N+M}c\left(k\right)e^{j2\pi k\Delta ft}\right]\times g\left(t\right)\times e^{j2\pi f_{c}t}\right\}$$

and $t\in \left[0,T+T_{g}\right],$ where T is the data symbols’ duration; $T_{g}$ is the guard interval duration; and $g\left(t\right)=1,\;t\in \left[0,T\right]$. Through its transmission over the UWA channel, the transmitted signal is corrupted due to the underwater channel multipath, ambient noise, Doppler shift, and additive white Gaussian noise (AWGN). The TDS-OFDM-NOMA is contentious for two branches at the receiver side, the energy harvesting part, and the information decoding part. These two parts will be explained in detail in separated sections below.

### 3.1. Underwater Acoustic Channel Model

The UWA channel is considered a time-variant linear system with a time-varying impulse response of $h\left(t,\tau \right)$; this impulse response describes the Doppler spreads and the channel multipath. For the UWA time-varying channel, the noise-free signal at the receiver side is described as a convolution of the channel impulse response and transmitted signal $s\left(t\right)$. In UWA communications, there are different models of the UWA impulse response $h\left(t,\tau \right)$ used to represent the UWA channel. The discrete multipath component models the UWA channel as [1,25]:

(12) $$h\left(t,\tau \right)=\sum_{p=1}^{L}A_{p}\left(t\right)\delta \left(\tau -\tau_{p}\left(t\right)\right)$$

where $A_{p}\left(t\right)$ and $\tau_{p}\left(t\right)$ are time-varying delays and amplitudes; L is the maximum length of the multipath components. $\delta \left(t\right)$ is the Dirac delta function. In this paper, we assume, $\tau_{p}\left(t\right)=\tau_{p}-𝒶t;$ hence, the channel model affects the received signal with a dilation factor a, and that factor is the same for all UWA channel multipaths. The received data frame over the UWA multipath channel can be represented as follows:

(13) $$y\left(t\right)=\overset{\infty }{\underset{-\infty }{\int }}h\left(t,\tau \right)s\left(t-\tau \right)d\tau$$

The received signal in the passband can be written as:

(14) $$\tilde{y}\left(t\right)=Re\left\{\sum_{p}A_{p}\left[\frac{1}{\sqrt{N}}\sum_{k=0}^{N-1}s\left(k\right)e^{j2\pi k\Delta ft\left(t+𝒶t-\tau_{p}\right)}+\sum_{k=N}^{N+M}\frac{1}{\sqrt{M}}\sum_{k=N}^{N+M}c\left(k\right)e^{j2\pi k\Delta ft}\right]\times \;g\left(t+𝒶t-\tau_{p}\right)\times e^{j2\pi f_{c}\left(t+𝒶t-\tau_{p}\right)}\right\}+\tilde{n}\left(t\right)$$

where $\tilde{n}\left(t\right)$ represents the total UWA channel noise; this noise includes the additive noise and the ambient noise. The received signal in the baseband version is satisfied by $\left\{\tilde{y}\left(t\right)\times e^{j2\pi f_{c}t}\right\}$ and can be written as:

(15) $$Y\left(t\right)=\frac{1}{\sqrt{N}}\sum_{k=0}^{N-1}\left\{s\left(k\right)e^{j2\pi k\Delta ft}e^{j2\pi 𝒶f_{k}t}\times \left[\sum_{p}A_{p}e^{-j2\pi f_{k}\tau_{p}}g\left(t+𝒶t-\;\tau_{p}\right)\right]\right\}+\frac{1}{\sqrt{M}}\sum_{k=N}^{N+M}\left\{c\left(k\right)e^{j2\pi k\Delta ft}e^{j2\pi 𝒶f_{k}t}\times \left[\sum_{p}A_{p}e^{-j2\pi f_{k}\tau_{p}}g\left(t+𝒶t-\tau_{p}\right)\right]\right\}+\tilde{n}\left(t\right)$$

Below, the acoustic propagation pass loss and the ambient noise will be addressed in detail. Over the UWA channel, each path $T\sim \frac{T}{\left(1+𝒶\right)}$. Each subcarrier practices a Doppler-made frequency shift $e^{j2\pi 𝒶f_{k}t}$, and it depends on the frequency of the subcarrier. The frequency-dependent Doppler shifts present severe intercarrier interference if an inefficient Doppler reparation is not achieved before the OFDM demodulation.

### 3.2. Acoustic Propagation Loss

The acoustic transmitted signal loss over a communication distanced b with carrier frequency f in dB over UWA communication in the ocean environments can be calculated as [26]:

(16) $$10\log PL\left(b,f\right)=𝓀.10\log b+b\;.10\log\alpha$$

where geometric spreading factor 𝓀 is 𝓀=1 and 𝓀=2 for shallow and deep water, respectively. α in dB/km is the absorption coefficient factor, and it can be written as [26]:

(17) $$\alpha =\frac{A_{1}P_{1}f_{1}f^{2}}{f^{2}+f_{1}^{2}}+\frac{A_{2}P_{2}f_{2}f^{2}}{f^{2}+f_{2}^{2}}+A_{3}P_{3}f^{2}$$

The restrictions given in (17) can be categorized as boric acid parameters, i.e., $f_{1}$, $A_{1}$ and $P_{1}$, magnesium sulfate parameters, i.e., $f_{2}$, $A_{2}$ and $P_{2}$, and pure water parameters, i.e., f, $A_{3}$ and $P_{3}$. For boric acid parameters, i.e., $f_{1}$, $A_{1}$ and $P_{1}$, $f_{1}$ is the boric acid relaxation frequency in kHz, and it can be written as [26]:

(18) $$f_{1}=2.8\;{\left(\frac{S}{35}\right)}^{0.5}\times 10^{\left[4-1245/\left(273+T\right)\right]}$$

where S is the water salinity in parts/1000, and T is the water temperature at °C. $A_{1}$ is the boric acid rate, and it is dependent on pH and the acoustic propagation speed of the ocean water obtained as [26]:

(19) $$A_{1}=\frac{8.68}{C}10^{\left(0.78\;\mathrm{pH}-5\right)}$$

$P_{1}=1\;$ is the pressure at a given depth in ocean water.

For the magnesium sulfate parameters, i.e., $f_{2}$, $A_{2}$ and $P_{2}$, $\;f_{2}$ is the magnesium sulfate relaxation frequency in kHz and is obtained as [26]:

(20) $$f_{2}=\frac{8.17\times 10^{\left[8-1990/\left(273+T\right)\right]}}{1+0.0018\left(S-35\right)}$$

$A_{2}$ is the value of the magnesium sulfate and obtained as [26]:

(21) $$\;A_{2}=21.44\frac{S}{C}\left(1+0.025T\right)$$

The depth pressure of the magnesium sulfate, $P_{2}$, is obtained by [26]:

(22) $$P_{2}=1-1.37\times 10^{-4}z+6.2\times 10^{-9}z^{2}$$

where z is the water depth in meters. 

$\;A_{3}$ is the viscosities of the pure water and can be obtained [26]:

(23) $$A_{3}=\left\{\begin{array}{c} \begin{array}{c} 4.937\times 10^{-4}-2.59\times 10^{-5}T+9.11\times 10^{-7\;}T^{2}-1.5\times 10^{-8}T^{3}\;\\ \;\mathrm{for}\;T\leq 20\;{}^{\circ}C \end{array}\\ \begin{array}{c} 3.964\times 10^{-4}-1.146\times 10^{-5}T+1.45\times 10^{-7}T^{2}-6.5\times 10^{-10}T^{3}\\ \;\mathrm{for}\;T>20\;{}^{\circ}C\; \end{array} \end{array}\right.$$

$P_{3}$ is the depth pressure of pure water and is written as [26]:

(24) $$P_{3}=1-3.83\times 10^{-5}z+4.9\times 10^{-10}z^{2}$$

### 3.3. Ambient Noise

The main source of noise in the UWA channel is ambient noise. This ambient noise is mainly dependent on turbulence, wind waves, thermal noise, and shipping. The power spectral density (PSD) of these sources of ambient noise is empirically formulated in dB re µPa/Hz as [26]:

(25) $$10\log N_{t}\left(f\right)=17-30\log f\;$$

(26) $$10\log N_{s}\left(f\right)=40+20\left(s-0.5\right)+26\log f-60\log\left(f+0.03\right)$$

(27) $$10\log N_{w}\left(f\right)=50+7.5\;w^{\frac{1}{2}}+20\log f-40\log\;\left(f+0.4\right)$$

(28) $$10\log N_{th}\left(f\right)=-15+20\log f$$

(29) $$N\left(f\right)=N_{t}\left(f\right)+N_{s}\left(f\right)+N_{w}\left(f\right)+N_{th}\left(f\right)$$

where $N_{t}$ is the PSD of the turbulence noise at (f<10 Hz). $N_{s}$ is the PSD of the shipping noise at (10 Hz~100 Hz) for a shipping activity factor, 𝓈=0, and 𝓈=1 for low and high activity, respectively. $N_{w}$ is the PSD of noise for the wind-driven wave at $\left(100\;\mathrm{Hz}\sim 100\;\mathrm{kHz}\right)$, and w is a m/s wind speed. $N_{th}$ is the PSD of the thermal noise, and it is the dominant one for f>100 kHz.

## 4. Energy Harvesting for TDS-OFDM-NOMA

**Figure 4:**
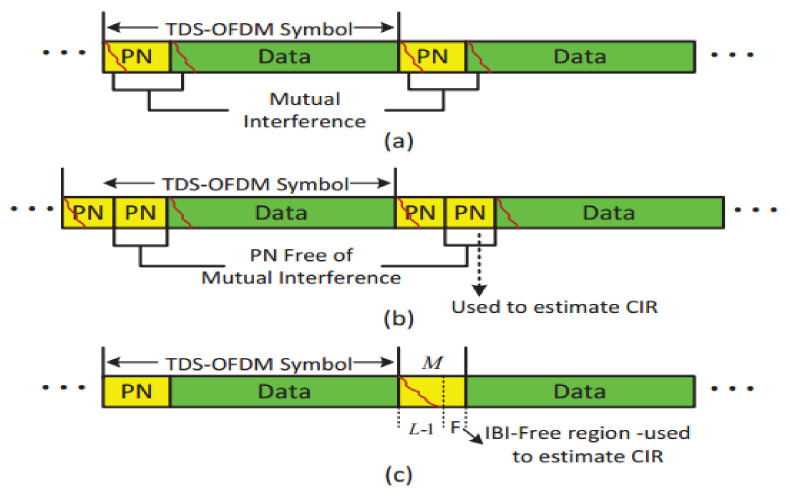
Received signal frame structure. (**a**) Conventional TDS-OFDM, (**b**) DPN-TDS-OFDM, (**c**) CS based TDS-OFDM.

In general, the UWA channel is a long-tap delay channel, and a long guard interval is required between transmitted OFDM blocks to avoid intercarrier interference (ICI); plus, a long pilot signal is needed to estimate such a channel type. To save the battery power of the underwater nodes, recently researchers preferred the TDS-OFDM as a fit candidate for underwater multicarrier modulation, especially with compressive sensing-based channel estimation to estimate the UWA channel impulse response. The small free region of the guard interval unaffected by the IBI between the OFDM data blocks and the training sequence is used for channel estimation purposes. In Figure 4, the received signal frame structure of different TDS-OFDM schemes is shown. To the best of our knowledge, all research has been focused on removing mutual interference between the OFDM data blocks and the PN training sequence for better synchronization and channel estimation. In addition, no one is working on saving energy from the received PN sequence. In this paper, we plan to use the wasted power of corrupted parts in guard intervals for energy harvesting. This paper works on energy harvesting for the underwater node using SWIPT in the time switching energy harvesting mode.

**Figure 5:**
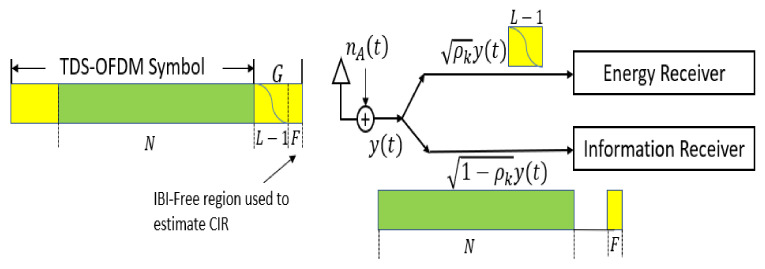
Architecture for the proposed energy harvesting receiver in IoUTs nodes based on TDS-OFDM communication system.

In this paper, the TS energy harvesting system is proposed to be used for the TDS-OFDM-NOMA system as shown in Figure 5. As shown in Figure 5, with the TS-mode, the first N data symbols, all the received power will be used for information decoding. As this OFDM subcarrier is carrying the data information, all signal power received is sent to the information receiver to achieve acceptable BER performance. For the residual OFDM frame, M PN symbols will be divided into two parts, the first part, the corrupted part L−1; in this part, all signal power will be used for energy harvesting, as it is less information, and it highly corrupted by the OFDM data block. In the second part of the received guard interval (the remaining uncorrupted part), $F=M-\left(L-1\right)$, all signals will be used for information decoding, and they will be used for channel estimation and equalization. Thus, the TS energy harvesting mode can be used in this paper as:

(30) $$\rho_{k}=\left\{\begin{array}{c} 0,\;k=1,\;\cdots \cdots ,N\;\\ 1,\;k=N+1,\;\cdots \cdots ,\;N+L-1\\ 0,\;k=N+L,\;\cdots \cdots ,\;N+M\; \end{array}\right..\;$$

In this way, the wasted power of the long guard interval (nearly 25% of OFDM transmitted power is wasted in guard interval) can be used in harvesting energy to increase the lifetime of the Internet of Underwater Thighs (IoUTs) nodes. The total energy harvested based on the proposed architecture can be written as:

(31) $$EH_{TDS}=\frac{T_{c}\eta Ps{\left|h\right|}^{2}}{2\left(1+b^{\alpha }\right)}$$

where $T_{c}$ is the period of the corrupted PN sequence, and η is the energy harvesting coefficient. Ps is the sink node transmitted power; *b* is the distance between the transmitter and receiver; and α is the underwater exponent path loss.

As shown in Figure 5, the energy receiver will be dialed with the corrupted part of the received PN sequence. On the receiver side, we consider the case where the receiver is solely decoding the information and harvesting the energy from the received signal simultaneously.

The received acoustic signal, $y\left(t\right)$, is first converted to a complex baseband signal $y_{b}\left(t\right)$ and then sampled and digitalized by an analog-to-digital converter for further decoding.

The noise introduced by the in-band harmonic distortion introduced by the RF band to based band signal conversion $n_{cov}\left(t\right)$ has $n_{cov}\sim CN\left(0,\sigma_{cov}^{2}\right)$. In our simulation and for simplicity, we assume an ideal ADC without any noise. With this assumption, the underwater acoustic channel can be given by $\;Y=\sqrt{hP}X+Z$, where *Y* and *X* denote the channel input and output, respectively, and $Z\sim CN\left(0,\sigma_{A}^{2}+\sigma_{cov}^{2}\right)$. In the energy part of the receiver, we harvest the energy from the received wireless signal. The received signal $y\left(t\right)$ is converted to be a direct current signal $i_{DC}\left(t\right)$ by a rectifier, $i_{DC}\left(t\right)$; this rectifier can be implemented by a Schottky diode and a passive low-pass filter (LPF). The direct current is then used to charge the battery and store the energy.

## 5. Information Receiver for TDS-OFDM-NOMA

The interference of the TDS-OFDM system has different features, as the OFDM data block is unknown, so it is difficult to get perfect symbols’ detection over the UWA channel as shown in Figure 4. In the TDS-OFDM, mitigation of the IBI represents a big challenge even with perfect channel knowledge. Removing the IBI of the TDS-OFDM data blocks into the training sequence is an uneasy task, as the receiver does not know the transmitted data of the OFDM blocks. On the contrary, the receiver knows the transmitted PN sequence; hence, by knowing the communication channel, removing the IBI of the PN sequence from the OFDM data block is an easy task.

The channel estimation task was a hard job to be estimated based on the corrupted PN sequence; hence many schemes have been proposed to solve this issue. The DPN-OFDM [21], ZPN-OFDM [22], and channel estimation based on the free IBI region of the received PN sequence were proposed to solve the UWA channel estimation problem. The DPN-OFDM is used twice as the guard interval to avoid the IBI of the OFDM blocks on the received PN sequence. Unfortunately, duplicated guard intervals waste the spectral and energy efficiency of the communication system; these cannot be accepted in underwater communication, as the energy and recharging capability of the underwater node is unaccepted. ZPN-OFDM was proposed to solve the energy efficiency problem of the DPN-OFDM, but, unfortunately, it fails to solve the wasted spectral efficiency problem. Recently, the IBI-free region of the received PN sequence was proposed to be used for channel estimation and equalization of the UWA TDS-OFDM communication system. In the same line, we will use the IBI-free region for compressive-sensing-based channel estimation. The small IBI free region is existing in the received PN training sequence in practical applications due to the system margin design, and the worst case happens when the guard interval length is equal to or less than the UWA channel length. To deal with that, we assume that the channel length is always less than the guard interval and that there is a free IBI region in the received PN sequence.

### 5.1. UWA Channel Estimation

The perfect channel estimation can provide a perfect removal of the IBI caused by the PN on the OFDM data blocks. After subtracting the IBI from the received OFDM data blocks, the TDS-OFDM will be equivalent to the BER performance of the conventional CP-OFDM and ZP-OFDM schemes. The differences in the received frame between the conventional TDS-OFDM, DPN-OFDM, and the CS-based OFDM used in this paper are shown in Figure 4.

As shown in Figure 4, the received PN sequence of the TDS-OFDM schemes is affected by the IBI because of the OFDM data blocks; this mutual interference is due to the UWA channel multipath. This mutual interference affected the accuracy of the UWA channel estimation. The time-domain compressive sensing channel estimation technique for the TDS-OFDM by using the received PN sequence can be written as follows [7]:

(32) $$z_{i}=\Psi_{i}h_{i}+v_{i}$$

where $v_{i}$ is the cumulative channel noise, and $\Psi_{i}$ is the sensing matrix used for compressive sensing. In this paper, the channel estimation is constricted based on the received PN sequence, and it can be written as:


(33) $$\Psi_{i}={\left[\begin{array}{cccc} c_{i,0} & x_{i-1,N-1} & \cdots & x_{i-1,N-L+1}\\ c_{i,1} & c_{i,0} & \cdots & x_{i-1,N-L+2}\\ \vdots & \vdots & \ddots & \vdots \\ C_{i,L-1} & C_{i,L-2} & \cdots & C_{i,0}\\ C_{i,L} & C_{i,L-1} & \cdots & C_{i,1}\\ \vdots & \vdots & \ddots & \vdots \\ C_{i,M-1} & C_{i,M-2} & \cdots & C_{i,M-L} \end{array}\right]}_{M\times L}$$

In the compressive sensing theory, the propagation paths P are affected by the sparsity level indication of the communication channel. For accurate channel estimation, the sparsity channel indicator should be small. The sparsity channel indicator is affected not only by the number of channels taps P; it is also affected by the channel length L and observation F, which is the number of rows. According to research work in [7], the sparsity indicator of the channel must satisfy the restricted isometry principle less (RIPless) condition for accurate channel estimation, and it can be represented in mathematical form as:

(34) $$P\leq \frac{F}{4{ℭ}_{0}\left(\log\left(\frac{L}{F}\right)+1\right)}$$

where ${ℭ}_{0}$ is a universal constant approximated by one, and it is normally adjusted according to the various situations of each compressive sensing matrix.

In the TDS-OFDM (*i* − 1)th received frame, the compressive sensing matrix in Equation (33) is represented the previously transmitted block. The previously transmitted block in (33) interferes with the current TDS-OFDM symbols, while the previous TDS-OFDM data block does not contain the last samples of the received training sequence M−L+1. In the compressive sensing-based IBI-free region, the last samples of the received PN training sequence are used for channel estimation, and the last F=M−L+1 rows of the $\Psi_{i}$ used as in [7] for channel estimation as an observation matrix can be written as follows:

(35) $$\Phi_{i}={\left[\begin{array}{ccccc} c_{i,L-1} & c_{i,L-2} & c_{i,L-3} & \cdots & c_{i,0}\\ c_{i,L} & c_{i,L-1} & c_{i,L-2} & \cdots & c_{i,1}\\ \vdots & \vdots & \vdots & \ddots & \vdots \\ c_{i,M-1} & c_{i,0} & c_{i,M-3} & \cdots & c_{i,\;M-L} \end{array}\right]}_{F\times L}$$

The observation matrix size F×L is determined by the received PN sequence. In the end, the compressive sensing equation of the free IBI region used in channel estimation will only be corrupted by the noise and can be written as follows:

(36) $$y_{i}=\Phi_{i}h_{i}+v_{i}$$

without any loss of generality, in this paper, we will apply the look-ahead backtracking orthogonal matching pursuit (LAB-OMP) algorithm proposed in [7] for channel estimation.

### 5.2. Doppler-Shift Compensations Using Two-Step Approach

In this paper, the two-step approach is used to compensate for the Doppler shift as in [25]. The doppler shift compensations based on the two-step approach are based on two points; (1) resampling for nonuniform Doppler compensation by converting the wideband into narrowband; (2) high-resolution uniform compensation of residual Doppler to avoid intercarrier interference. For the resampling method in the passband signals, the received signal is resampled using a resampling factor 𝒷 as $\tilde{z}\left(t\right)=\tilde{y}\left(\frac{t}{1+𝒷}\right)$. The effect of the resampling will be in two directions, rescaling the waveform and providing a frequency-dependent Doppler compensation. After resampling the received baseband signal, it can be written as:

(37) $$z\left(t\right)=e^{j2\pi \frac{𝒶-𝒷}{1+𝒷}f_{c}t}\times \left\{\frac{1}{\sqrt{N}}\sum_{k=0}^{N-1}\left\{s\left(k\right)e^{j2\pi k\Delta f\frac{1+𝒶}{1+𝒷}t}\times \left[\sum_{p}A_{p}e^{-j2\pi f_{k}\tau_{p}}g\left(\frac{1+𝒶}{1+𝒷}t-\;\tau_{p}\right)\right]\right\}+\frac{1}{\sqrt{M}}\sum_{k=N}^{N+M}\left\{c\left(k\right)e^{j2\pi k\Delta f\frac{1+𝒶}{1+𝒷}t}\times \left[\sum_{p}A_{p}e^{-j2\pi f_{k}\tau_{p}}g\left(\frac{1+𝒶}{1+𝒷}t-\tau_{p}\right)\right]\right\}+v\left(t\right)\right\}$$

where $v\left(t\right)$ is the ambient and additive noise. If $\frac{1+𝒶}{1+𝒷}$ is close to one, then the received signal can be as:

(38) $$z\left(t\right)\approx e^{j2\pi \frac{𝒶-𝒷}{1+𝒷}f_{c}t}\times \left\{\frac{1}{\sqrt{N}}\sum_{k=0}^{N-1}\left\{s\left(k\right)e^{j2\pi k\Delta ft}\times \left[\sum_{p}A_{p}e^{-j2\pi f_{k}\tau_{p}}g\left(t-\tau_{p}\right)\right]\right\}+\;\frac{1}{\sqrt{M}}\sum_{k=N}^{N+M}\left\{c\left(k\right)e^{j2\pi k\Delta ft}\times \left[\sum_{p}A_{p}e^{-j2\pi f_{k}\tau_{p}}g\left(t-\tau_{p}\right)\right]\right\}+v\left(t\right)\right\}$$

The residual Doppler effect is the same over all the subcarriers; hence, the wideband OFDM system is converted into a narrowband OFDM system with a frequency-independent Doppler shift, representing the carrier frequency offset (CFO), $\epsilon =\frac{𝒶-𝒷}{1+𝒷}f_{c}$. By estimating the CFO, the received signal can be compensated. The Doppler scaling factor estimation and frame synchronization can be based on the preamble and the post-amble of a data packet as in [24,25,27,28].

### 5.3. Frame Synchronization and Coarse Doppler Scaling Factor Estimation

The time synchronization and Doppler scaling Factor (DSF) estimation have a lot of attention on underwater research spatially in early underwater research work. The most common way for synchronization and channel estimation is to exploit an embedded repetitive pattern in the transmitted preamble as reported in [24,27,28], and we use the same way for DSF estimation and time synchronization. The corresponding P-tap window correlation can be written as:

(39) $$\left({\hat{P}}_{i},{\hat{a}}_{i}\right)=\arg\underset{P,a}{\underset{︸}{\max\;}}\frac{\sum_{𝓅=a}^{a+P-1}z_{i,𝓅}^{*}z_{i,𝓅+P}}{\sqrt{\sum_{𝓅=a}^{a+P-1}{\left|z_{i,𝓅}\right|}^{2}\sum_{𝓅=a}^{a+P-1}{\left|z_{i,𝓅+P}\right|}^{2}}}$$

where ${\hat{a}}_{i}$ is the received preamble starting time. ${\hat{P}}_{i}$ is an integer representing the coarse estimation of the initial tap delay, $\beta_{i,0}$, discussed in detail in [24,27,28], and the estimated initial tap delay can be written as follows:

(40) $${\bar{\beta }}_{i,0}=\frac{\alpha N-{\hat{P}}_{i}}{{\hat{P}}_{i}}$$

The estimated initial tap delay in (40) offers the frame time synchronization. The DSF estimation is limited by integer parameter α. The estimated Doppler shift of the UWA channel can be written as:

(41) $$\in_{i,0}=\frac{\beta_{i,0}-{\bar{\beta }}_{i,0}}{1+{\bar{\beta }}_{i,0}}\times \frac{F_{c}}{B}$$

## 6. Experimental Results and Discussion

**Figure 6:**
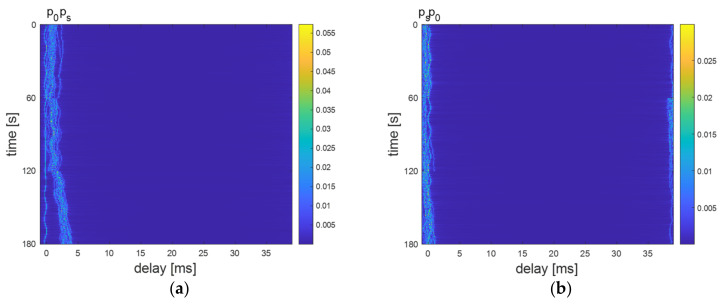
The time evolution of the magnitude baseband impulse response for (**a**) near UUV at distance 100 m, (**b**) far UUV at distance 800 m.

In this section, we will deal with the simulations carried out on MATLAB to evaluate the performance of the proposed TDS-OFDM-NOMA compared with the conventional CP-OFDM-NOMA in terms of BER and the system throughput. Moreover, in this section, we will evaluate the performance of the proposed energy harvesting system for the TDS-OFDM scheme over the long UWA tap delay channel. The sink node generates $10^{6}$ random information bits and transmits to far and near users over the time-varying UWA channel. The simulation parameters of this paper are shown in Table 1. The underwater path loss is obtained by using Equation (16). In this paper, we use a Gaussian assumption geometric description for the underwater acoustic channel model to evaluate the proposed TDS-OFDM-NOMA scheme [29]. This underwater acoustic model reflects the physical features of the acoustic propagation and the predictable random channel disparities. The far and near users are dropped randomly in the simulation area. Based on the distances between the users and the sink nodes, the underwater acoustic channels are recognized. The underwater acoustic channel parameters for near and far users are listed in Table 2, and the time development of the extent of the baseband impulse response for rapid underwater acoustic channels of the near and far user is shown in Figure 6a,b, respectively. In Figure 6, the distance between the transmitter and receiver is 50 m and 100 m for far and near users, respectively.

### 6.1. System Throughput Improvement

The main motivation of the proposed TDS-OFDM-NOMA scheme is to improve the UWA communication system throughput using only the OFDM multicarrier schemes exclusive to the resource allocation as a communication system treatment with both good and poor channel conditions in the same way.

While the NOMA scheme allows users to access the resource allocation regardless of their channel conditions, in the NOMA scheme, far and near users have the freedom to access all channel resources. Thus, the channel is utilized by users with both good and bad channel conditions. In this way, the limited frequency channel can be allocated at the same time to different users belonging to the same cell. TDS-OFDM-NOMA improves the overall system capacity. Using the TDS-OFDM scheme in the state of the conventional ZP-OFDM or CP-OFDM in the NOMA scheme improves the total system throughput. The TDS-OFDM improves the subcarrier utilization by avoiding a pilot signal in the transmitted subcarrier. Since, in our work, the spectral efficiency analysis is affected by the UWA multipath fading distortion, AWGN, and ambient noise, the signal-to-noise distortion (*SNDR*) can be represented in mathematical form as:

(42) $$SNDR=\log\left(\frac{P_{s}}{P_{N}+P_{D}}\right)$$

where $P_{s}$ is the signal power, while $P_{N}$ and $P_{D}$ denote the total underwater noise and the distortion power, respectively. In case there are two users only as we consider in the simulation of this paper, the achievable data rate of NU (*R*_1_) and FU (*R*_2_) in the presence of multipath distortion and underwater ambient noise can be explained as follows:

(43) $$R_{1}=\log_{2}\;\left(1+\frac{P_{1}{\left|h_{1}\right|}^{2}}{P_{D}+\chi_{1}+\rho_{1}}\right)$$

(44) $$R_{2}=\log_{2}\;\left(1+\frac{P_{2}{\left|h_{2}\right|}^{2}}{P_{1}{\left|h_{2}\right|}^{2}+P_{D}+\chi_{2}+\rho_{2}}\right)$$

where $\chi_{1}$ and $\chi_{2}$ represent the AWGN of the near and far users, respectively. Similarly, $\rho_{1}$ and $\rho_{2}$ are the ambient UWA channel noise of the NU and FU, respectively. $P_{1}{\left|h_{2}\right|}^{2}$ represents the effect of the NU with a better channel condition and the FU with a poor channel condition. The total system rate of the proposed OFDM-NOMA UWA system can be written as:

(45) $$R=R_{1}+R_{2}=\log_{2}\;\left(1+\frac{P_{1}{\left|h_{1}\right|}^{2}}{P_{D}+\chi_{1}+\rho_{1}}\right)+\log_{2}\;\left(1+\frac{P_{2}{\left|h_{2}\right|}^{2}}{P_{1}{\left|h_{2}\right|}^{2}+P_{D}+\chi_{2}+\rho_{2}}\right)$$

In conventional CP-OFDM and ZP-OFDM, a group of subcarriers is used for pilot signal transmission; these subcarriers represent a wasted resource. In contrast, all the TDS-OFDM subcarriers are used for data transmission. The subcarrier utilization of different OFDM can be written as follows:

1. The subcarrier utilization of the CP-OFDM and ZP-OFDM schemes can be written as:

(46) $$\xi_{1}=\frac{N_{Data}}{N_{Data+N_{Pilots}}}\times \frac{N}{N+M}$$

2. The subcarrier utilization of the TDS-OFDM can be written as:

(47) $$\xi_{2}=\frac{N}{N+M}$$

where $N_{Data}$ and $N_{Pilots}$ represent the number of OFDM subcarriers used for data transmission and the number of the OFDM subcarrier used for pilot signal transmission. The system throughput of the ZP-OFDM-NOMA and CP-OFDM-NOMA can be written as:

(48) $$\gamma_{1}=\xi_{1}R$$

and the system throughput of the TDS-OFDM-NOMA can be written as:

(49) $$\gamma_{2}=\xi_{2}R$$

**Figure 7:**
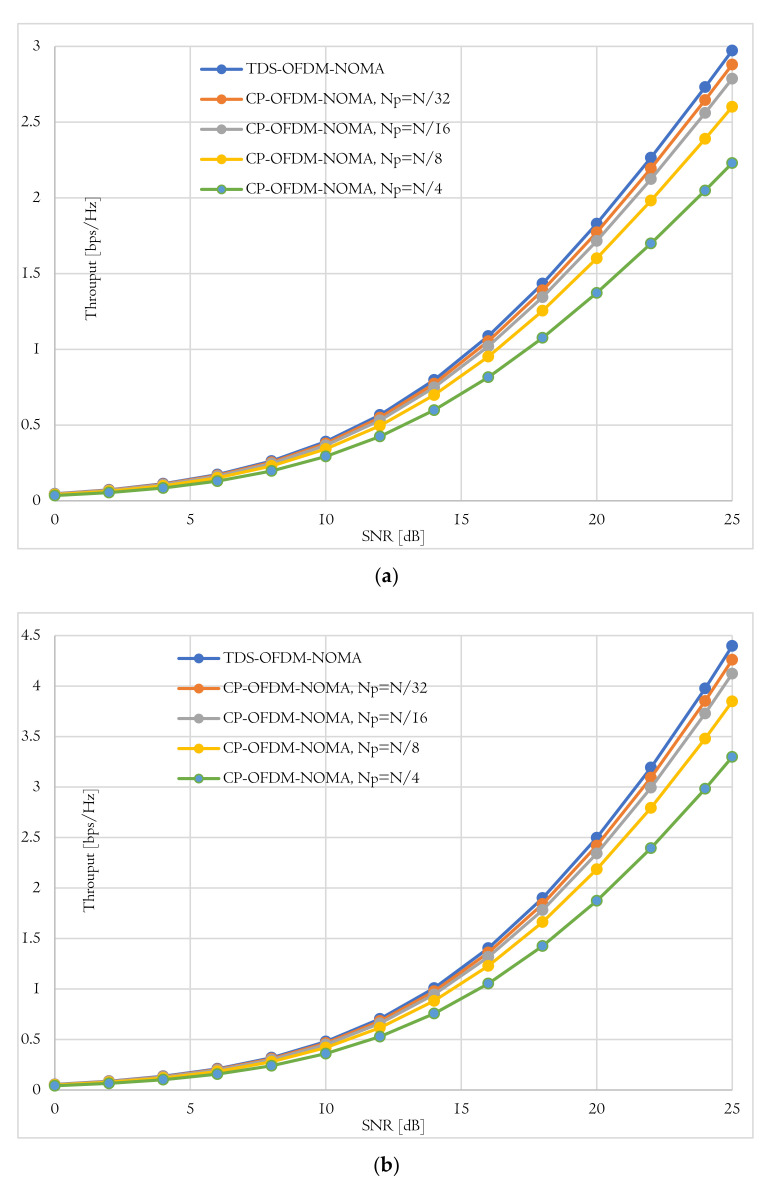
Average throughput achieved by TDS-OFDM-NOMA and CP-OFDM-NOMA under different pilots’ length for (**a**) Near user and (**b**) Far user.

Figure 7 shows the behavior of the total system throughput of the near and far user versus the SNDR in the case of using the proposed TDS-OFDM-NOMA scheme and conventional CP-OFDN-NOMA scheme with different pilots’ length. As known, as the pilot’s length increases, the total system throughput decreases. Thanks to the TDS-OFDM pilot’s freedom, the TDS-OFDM-NOMA provides the highest system throughput for the near and far users. At a SNDR of 20 dB, the near user of the TDS-OFDM-NOMA scheme achieved 2.33 bits per second per hertz (pbs/Hz), while the conventional CP-OFDM-NOMA achieves 2.26, 2.18, 2.04, and 1.75 pbs/Hz for the pilot’s length of N/32, N/16, N/8, and N/4, respectively. In the case of far user, at a SNDR of 20 dB, the TDS-OFDM-NOMA scheme achieved 2.14 pbs/Hz, and the convention CP-OFDM-NOMA achieved 2.07, 2, 1.87, and 1.6 bps/Hz for a pilot’s length equal to N/32, N/16, N/8, and N/4, respectively.

### 6.2. Analysis of Bit Error Rate

**Figure 8:**
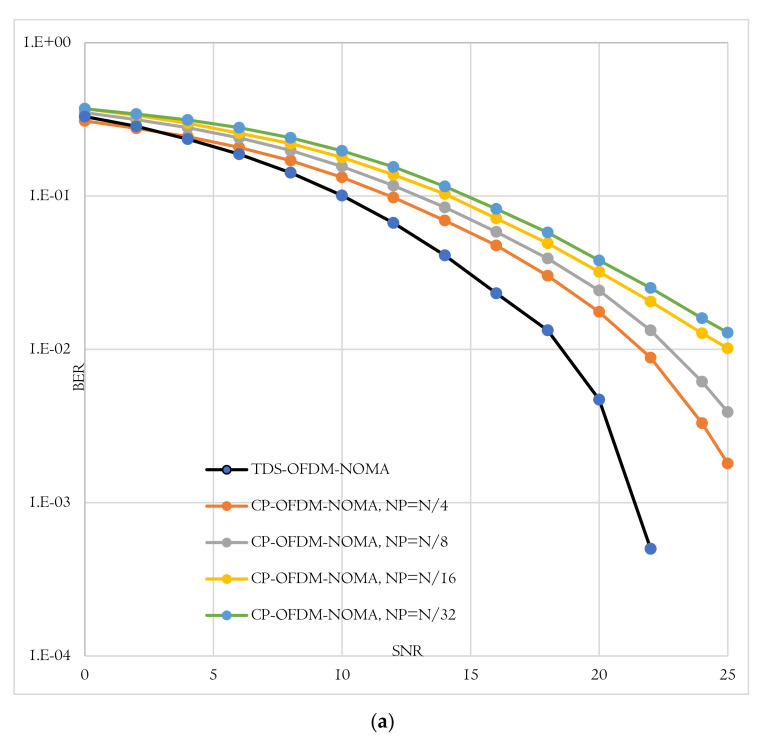

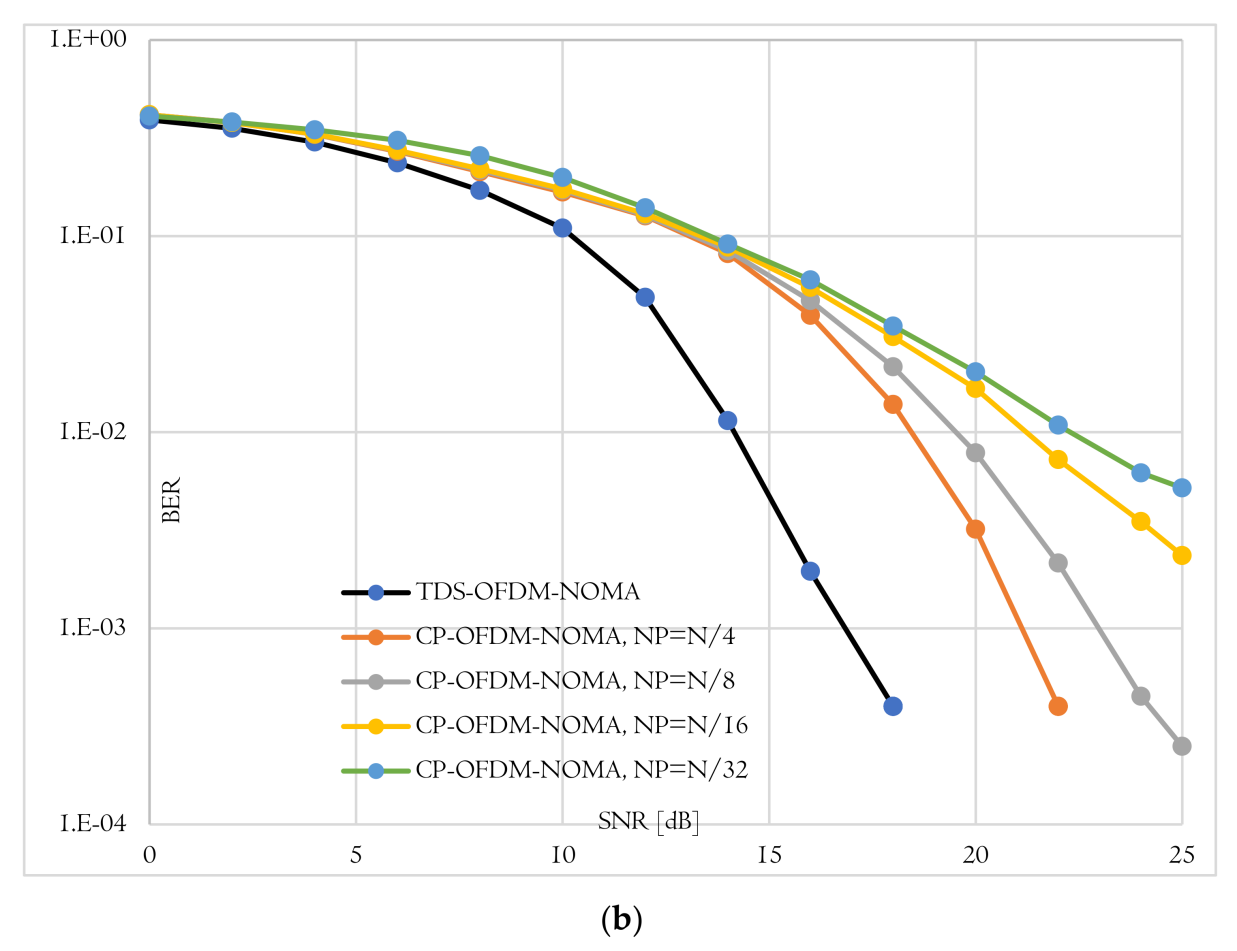
BER-total comparison of proposed TDS-OFDM-NOMA and convention CP-OFDM-NOMA; (**a**) Uncoded system and (**b**) Coded system.

**Figure 9:**
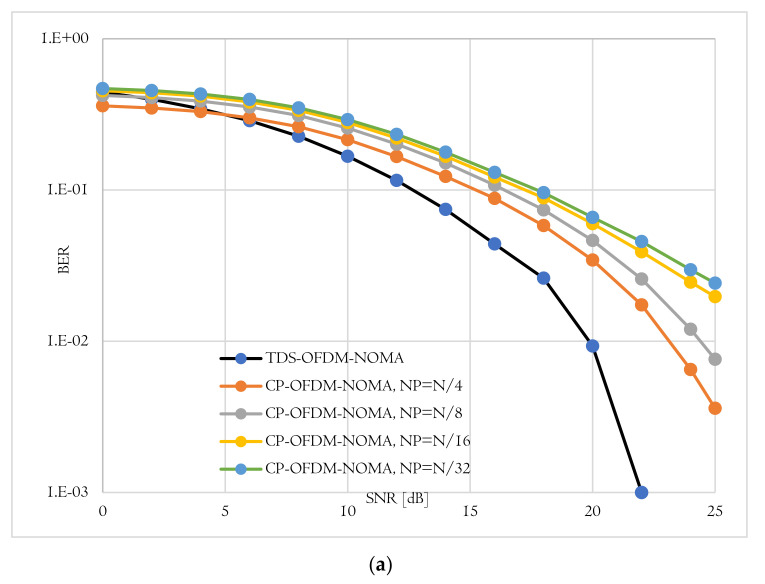

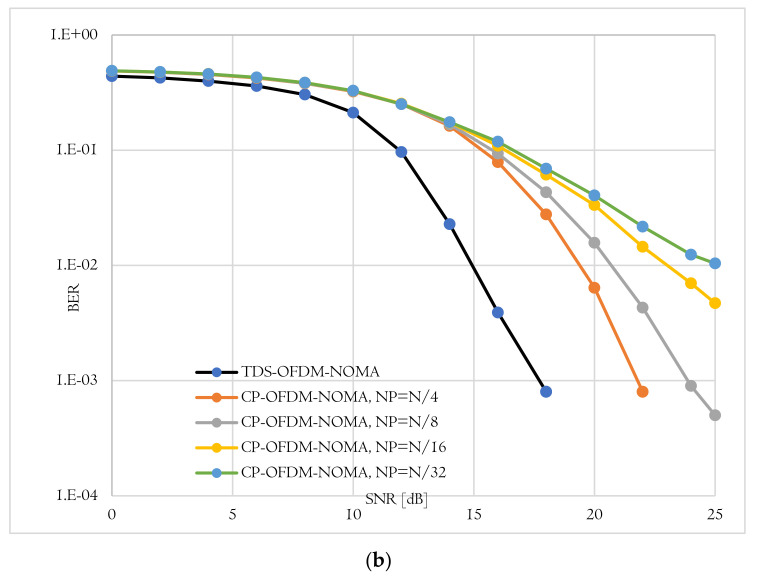
BER-(far-user) comparison of proposed TDS-OFDM-NOMA and convention CP-OFDM-NOMA; (**a**) Uncoded system and (**b**) Coded system.

**Figure 10:**
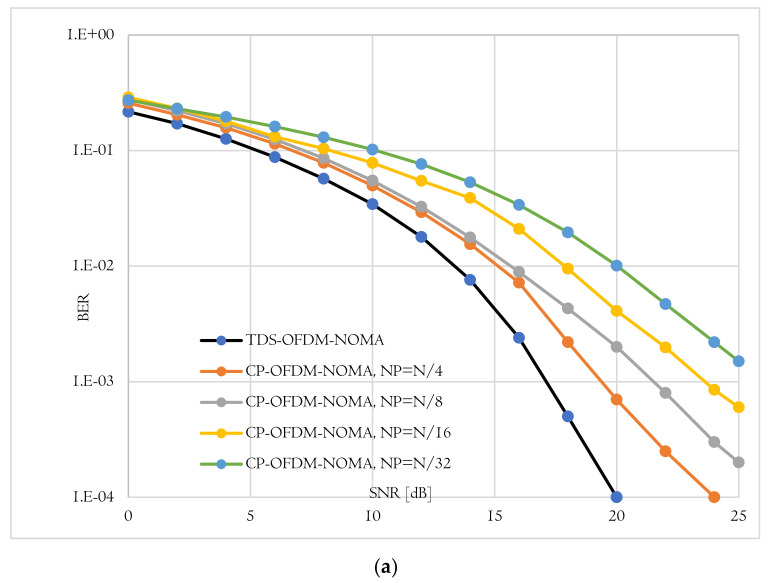

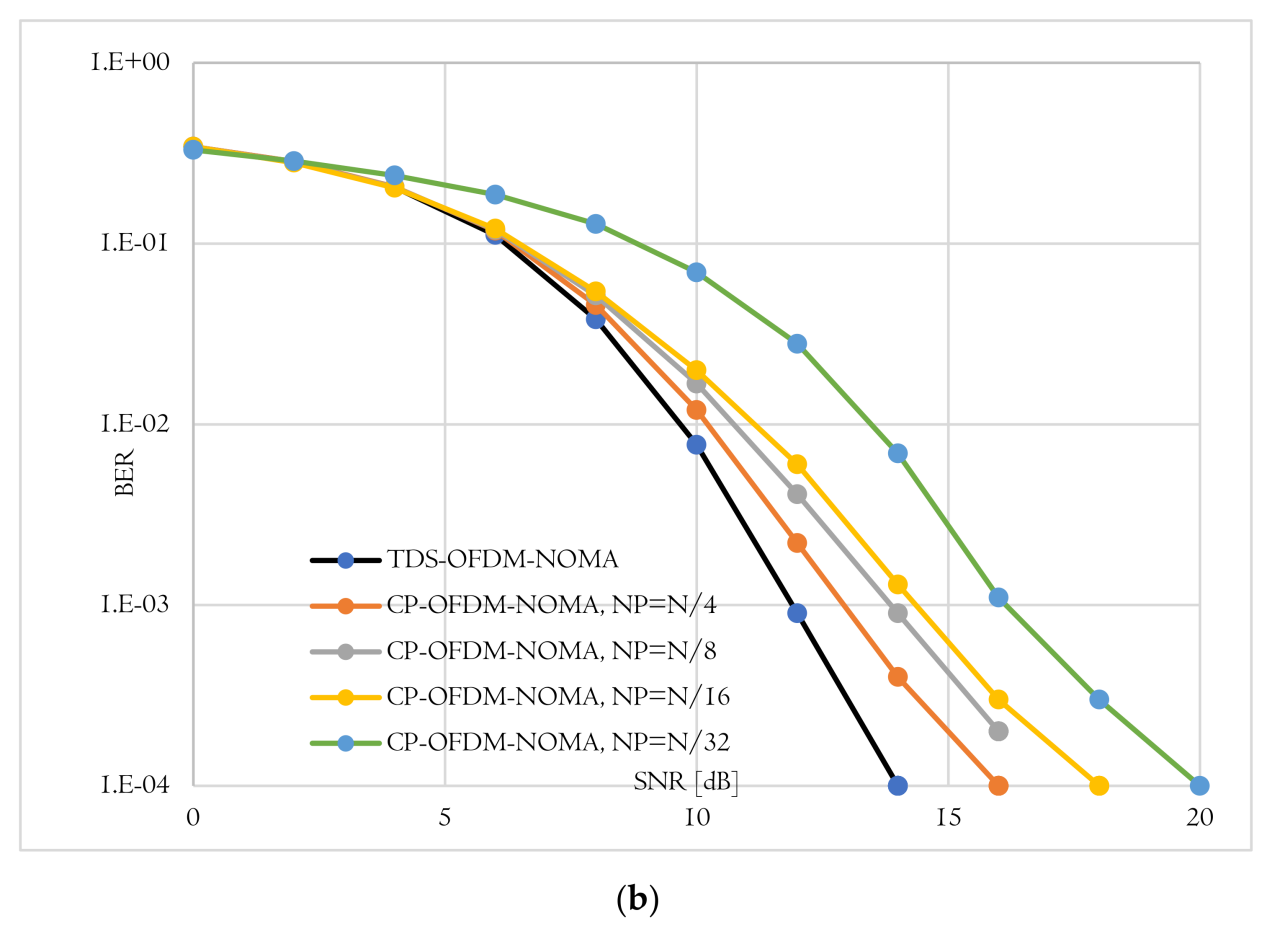
BER-(near-user) comparison of proposed TDS-OFDM-NOMA and convention CP-OFDM-NOMA; (**a**) Uncoded system and (**b**) Coded system.

The BER performance is the second evaluation metric used to evaluate the proposed TDS-OFDM-NOMA scheme over the underwater acoustic channel. In this evaluation, the proposed TDS-OFDM-NOMA, BER performance over the underwater acoustic channel is evaluated and compared to the conventional CP-OFDM-NOMA. Thanks to the IBI-free region compressive sensing channel estimation, the TDS-OFDM-NOMA provides a BER performance better than the performance of the conventional CP-OFDM-NOMA. In conventional CP- OFDM-NOMA, we use the least square method for channel estimation. The total BER performance of the proposed TDS-OFDM-NOMA system is studied by evaluating its uncoded and coded BER performance as shown in Figure 8, Figure 9 and Figure 10. In all of the simulation experiments, data symbols are created from random information bits which are first encoded by a rate 1/2 convolutional coder with the generator [65, 57] polynomial. For the coded case, the coded bits are interleaved by a block interleaver of depth 8 before QPSK modulation. Based on the BER metric, the proposed TDS-OFDM-NOMA scheme outperforms the conventional CP-OFDM-NOMA in total system BER (the average BER in NU and FU), the BER of the NU, and the BER of the FU. This BER improvement is due to efficient IBI removal from the TDS-OFDM data blocks thanks to the accurate channel estimation technique using the LABOMP compressive.

### 6.3. Energy Efficiency Improvement

In this sub-section, we evaluate the proposed energy harvesting scheme of the proposed TDS-OFDM-NOMA in two terms, energy efficiency and harvested energy. The energy efficiency of the TDS-OFDM can be formulated as:

(50) $$EE_{TDS-OFDM}=\frac{N}{N+\beta^{2}M}\times 100\%$$

where $\beta =\sqrt{2}$ is the amplitude factor clamped on the guard interval in the time domain. The proposed energy harvested TDS-OFDM scheme (EH-TDS) can be calculated as:

(51) $$EE_{EH-TDS}=\frac{N}{N+\beta^{2}\left(\gamma \left(L-1\right)+F\right)}\times 100\%$$

where $\gamma =\left(1-\frac{\eta {\left|h\right|}^{2}}{2PL}\right)$ is the efficiency factor for using the guard interval transmitted power in energy harvesting, and F=M−L+1 is the IBI free region length used in equalization and the channel estimation. The conventional TDS-OFDM has energy efficiency calculated by [7]:

(52) $$EE_{TDS}=\frac{N}{N+\beta^{2}M}\times 100\%$$

**Figure 11:**
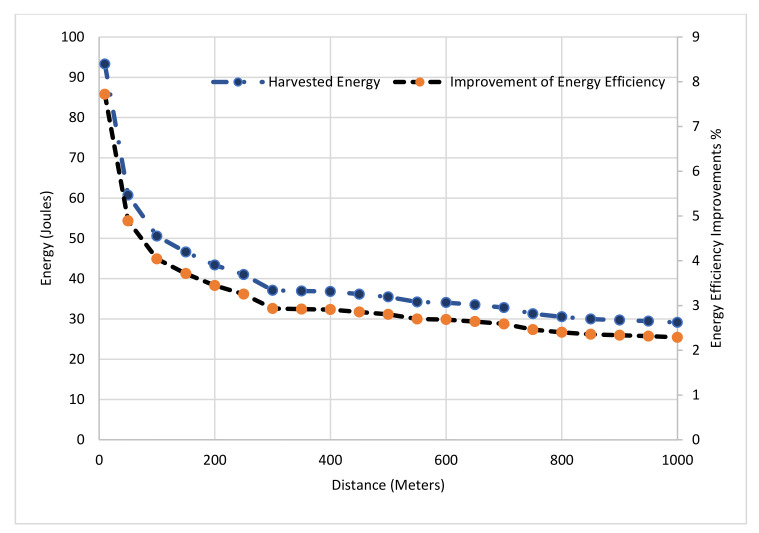
The performance of the TDS-OFDM using TS-SWIPT verses the communication distance, b.

The energy efficiency improvement, $\xi =EE_{EH-TDS}-EE_{TDS}$, as a function of transmitted distance, b is shown in Figure 11. We observe that the energy efficiency is decreased by increasing the transmission distance due to the UWA channel path loss. In Figure 11, the guard interval length is set to be M=255; the maximum UWA channel length is set to be L=180; and the IBI Free region length is F=75. The battery of the wireless sensor nodes can harvest at least 30 joules at each transmitted frame of the TDS-OFDM.

**Figure 12:**
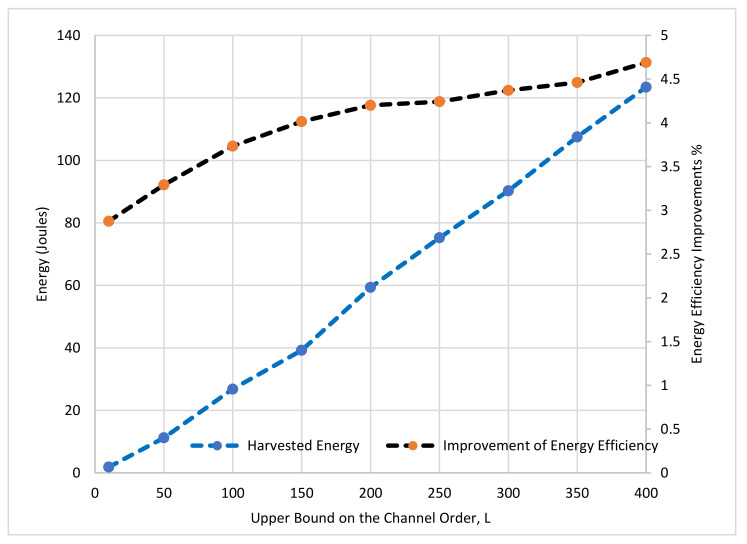
The performance of the TDS-OFDM using TS-SWIPT verses the maximum channel order, L.

The energy harvested improvement for TDS-OFDM can be reached up to 8% in a short communication distance. Figure 12 shows the performance of the TDS-OFDM over the UWA channel at different maximum channel tap delays, as the maximum channel tap delay increases; the improvement of the energy efficiency and the harvested energy both are increased using the proposed energy harvesting scheme where the duration of the symbols used in harvested energy is increased.

## 7. Conclusions

In this paper, a novel energy-harvested TDS-OFDM-NOMA scheme is introduced for UWA communication. The proposed energy harvested scheme uses the long UWA channel tap delay in harvesting energy using TS-SWIPT and increases the battery lifetime. The UWA TDS-OFDM-NOMA communication can efficiently harvest the energy via the long guard interval. By the proposed scheme, the drawback of the long guard interval used in UWA communication is converted to be useful in energy harvesting. On the other hand, TDS-OFDM-NOMA fulfills high throughput communication over the limited UWA channel bandwidth. It is observed that the proposed TDS-OFDM-NOMA performs better as compared with the conventional CP-OFDM-NOMA in the UWA channel. Results were driven in terms of BER, total system throughput, energy efficiency, and harvested energy.

## Figures and Tables

**Table 1:** Simulation Parameters.

Parameter	Value
Transmitted Power (Ps).	10 Watts
$\mathrm{Transmission}\;\mathrm{Period}\;\left(T\right).$	One second
Energy Harvesting Coefficient (η).	0.7
Power Allocation Coefficients, ${\left|p_{1}\right|}^{2}$.	0.6
Power Allocation Coefficients, ${\left|p_{2}\right|}^{2}$.	0.4
Carrier Frequency $\left(f\right)$	15 kHz
$\mathrm{Channel}\;\mathrm{Bandwidth}\;\left(B\right)$	6 kHz
Modulation order	2-QAM

**Table 2:** Parameters of the UWA channel model.

Parameters	Near Users (B)	Far Users (A)
Surface Height (m)	$\left(100∼50\right)$	$\left(50∼10\right)$
Tx Height (m)	1000	1000
Rx Height (m)	$\left(100∼50\right)$	$\left(50∼10\right)$
Channel Distances	$\left(10∼50\right)$	$\left(50∼100\right)$
Frequency Range (KHz)	$\left(10∼15\right)$	$\left(10∼15\right)$
Doppler Factor (v/c)	7 × 10^−2^	7 × 10^−2^

## Data Availability

The authors declare that the data used to support the findings of this study will be available from the corresponding author upon request.
